# Novel Use of a Tracheostomy Tie String to Secure an Endotracheal Tube in Procedural Areas

**DOI:** 10.7759/cureus.86730

**Published:** 2025-06-25

**Authors:** Matthew Weinger, Meredith Kingeter

**Affiliations:** 1 Anesthesiology, Vanderbilt University Medical Center, Nashville, USA

**Keywords:** adhesive tape, anesthesiology, endotracheal tube, intubation, tracheostomy tie

## Abstract

While seemingly straightforward, securing an endotracheal tube (ETT) or laryngeal mask airway (LMA) during an anesthetic is nuanced and complex. Most providers use various kinds of tape (silk, paper, or plastic) to secure the airway to the patient’s face to prevent the tube from becoming dislodged during the case, which would threaten the ability to oxygenate, ventilate, and anesthetize the patient safely. Many factors determine how an anesthesia provider chooses to secure the airway, including the patient’s body habitus, allergies, type and location of surgical procedure, and individual provider preference. This technical report describes a novel, safe, and alternative way of securing the ETT or LMA by using the tracheostomy (trach) tie string instead of tape or an industrial securement device.

## Introduction

Securing and maintaining an airway while a patient is under general anesthesia and unable to protect their own airway is a tenet of the field of anesthesiology. A large, closed claim review from 20 years ago revealed that inadequate ventilation/oxygenation and premature extubation were two of the four most common respiratory complications resulting in adverse outcomes; both of which can result from endotracheal tube (ETT) migration [1].

Around the same time, Dr. MBW anesthetized an older woman for a procedure. He secured the ETT with "pink tape" across her cheeks and upper lip. At the end of the case, when he removed the tape, a significant layer of skin came off with it, much to his and the patient’s dismay. This prompted him to wonder if there was a better way to secure a tube. After some trial and error, he settled on the use of 20 to 24 inches of ¼-inch soft cotton "trach tie" around the back of the neck, using a slide-and-grip hitch knot around the ETT at the lips.

He chose this knot over the previously documented clove or cow (or lanyard) hitch, which can be slower to perform, must pass over the tube, and is more difficult to loosen to adjust or remove if necessary [2]. Since the ties are cut to size from long rolls, the cost is comparable to tape while being appreciably less expensive than the many commercial devices designed specifically to secure ETTs with comparable levels of security from movement [3].

Dr. MBW has since used this technique in over 1500 cases for both ETTs and laryngeal mask airways (LMAs) without a single failure. He has taught a generation of residents and student nurse anesthetists to use this technique, including the co-author. The technique is most useful in patients with beards or skin to which tape will not stick (e.g., burns), and in situations where the ETT will need to move during the case (e.g., orally intubated dental cases). Moving the ETT from one side of the mouth to the other takes virtually no time (<4 seconds) with this tie method versus more than 30 seconds with untaping and re-taping [4].

## Technical report

**Figure 1:**
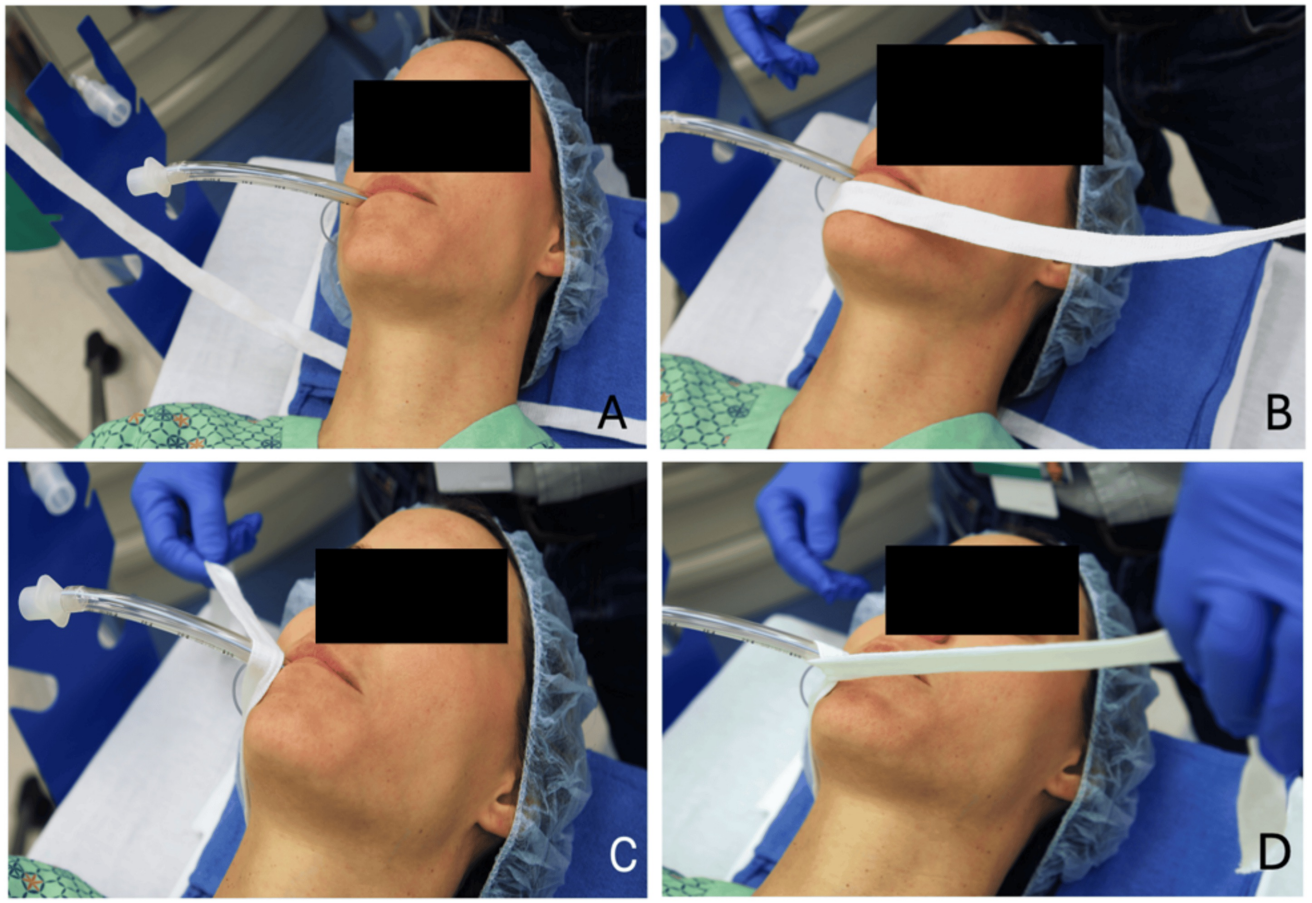
Sequence of steps (A-D) to tie an endotracheal tube (ETT). (A) Pre-cut the tie on the bed so it lies directly beneath the patient’s neck, with more length on the "right" side of the patient. (B) The right side of the tie length is brought up and under the ETT. (C) The tie length is brought around the ETT (counterclockwise from the foot of the bed) once. (D) The tie length is brought around the ETT a second time. The “patient” shown in the figure is a co-author, not a real patient; images are de-identified per author preference.

**Figure 2:**
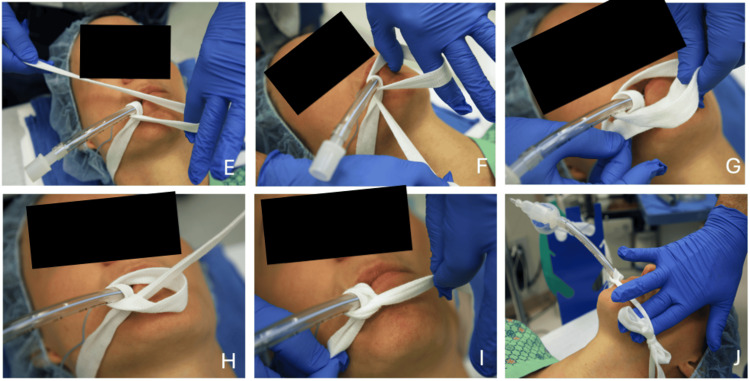
Sequence of steps (E-J) to tie an endotracheal tube. (E) On the second time around, place two fingers in the loop. (F) Continue to pass the tie length a second time. (G) The end is passed under the preceding loop. (H) The end is passed through the preceding loop to complete the slip knot. (I) The slip knot is pulled tight at the lips. (J) The tie is secured with a bow tie on the left cheek and is loose enough to allow two fingers to fit easily between the tie and either cheek. The “patient” shown in the figure is a co-author, not a real patient; images are de-identified per author preference.

At the start of the case, place the pre-cut tie on the bed so it lies directly beneath the patient’s neck (Figure 1A). For a right-handed clinician, it is best if two to three times as much of the tie is on the right side of the neck compared with the left. After intubation, the visible patient’s right side of the length is brought up, under (Figure 1B), and around (counterclockwise from the foot of the bed) the tube once (Figure 1C). On the second time around (Figure 1D), the end is passed under and through the preceding loop (Figures 2E-2G). The slip knot is pulled tight at the lips (Figures 2H-2I) and secured with a bow tie on the left cheek (Figure 2J). The loop around the neck should be “dog collar tight” (i.e., loose enough to allow two fingers to fit easily between the tie and either cheek). Video 1 illustrates the entire process.

**Video 1:** Securing the ETT with trach ties. ETT: endotracheal tube The “patient” shown in the video is a co-author, not a real patient; images are de-identified per author preference.

For extubation, simply untie the bow tie and remove the tube with the tie attached. In large-necked individuals, it is useful to pull the left side tie through behind the neck before removing the tube, as otherwise the tie can get stuck underneath the neck tissue before the tube is completely out of the mouth.

## Discussion

The simple trach tie method of securing an ETT is as secure as tape and is potentially more secure in situations where the patient’s skin is unavoidably wet or slick. Tape peeling off the face is the most common mechanism of ETT migration and failure, and is thus avoided in the case of tying [5]. It is quick and easy to tie, untie, and reposition, and unlikely to cause tissue injury.

There are many commercial ETT securing devices that can accomplish the same task. These are much more expensive than either this method or traditional taping. Therefore, they should be reserved for extended ETT securing, typically in the ICU or in prolonged operative procedures in patients with facial burns or secretions.

Commercial ETT securing devices have also been studied versus traditional taping methods and some tying methods, without a clear consensus on best methodology [3,4,6,7]. Many of these existing studies are performed in a simulation lab and translated to clinical care. Of the clinical data, most are retrospective from the ICU and not in the operating room, which confers a different set of conditions and requirements.

Caveats and limitations

Aside from inadvertent slippage and extubation, the primary risks of tying versus taping are diminished venous drainage from the head and, to a lesser extent, insufficient arterial flow to the head. This technique should not be used in pediatrics, since the millimeters of slack to allow for cerebral blood flow can cause significant ETT migration in small patients. Other contraindications for tying as opposed to taping a tube include (1) significant carotid artery insufficiency; (2) significant cerebral venous occlusion; (3) extant cerebral hypertension or edema; and (4) non-supine positioning (e.g., prone, lateral, extended Trendelenburg). Avoid tying the ETT for procedures lasting more than four hours, although this is a conservative recommendation. In all cases, it is important that the tie is not too tight; there should be no focal pressure on the neck skin.

Because the tie cannot be used in a sterile field, its use is precluded in many head, intracranial, facial, and neck cases. Even if a surgeon is amenable in such cases, it is advisable to avoid tube tying when there is going to be surgical head movement and the head is not readily available (e.g., head of bed turned 180°). When obtaining internal jugular venous access, one can slide the tie temporarily above the ear to avoid the sterile field of the central line. It can be more difficult to get a secure tie in patients who are edentulous, although keeping the tie on the mandible and securing the bow tie with a small piece of tape is often effective in such circumstances.

## Conclusions

Securing an ETT or LMA with trach tie string as an alternative to the traditional tape method is often a feasible, safe, easy, and cost-effective method that has not been widely discussed in the literature. The trach tie approach also minimizes skin damage that can be caused by taping. Notable contraindications to this approach include non-supine positioning, head or neck cases, and patients with the potential for brain, head, or neck edema. Further studies are required to examine the incidence of tape-induced skin damage, as well as providers’ opinions of this alternative approach to securing an ETT.
